# Trends and Obstacles to Implement Dynamic Perfusion Concepts for Clinical Liver Transplantation: Results from a Global Web-Based Survey

**DOI:** 10.3390/jcm12113765

**Published:** 2023-05-30

**Authors:** Alessandro Parente, Mauricio Flores Carvalho, Rebecca Panconesi, Yuri L. Boteon, Riccardo De Carlis, Philipp Dutkowski, Paolo Muiesan, Daniele Dondossola, Andrea Schlegel

**Affiliations:** 1 HPB and Transplant Unit, Department of Surgical Science, University of Rome Tor Vergata, 00133 Rome, Italy; aleparen@gmail.com; 2 Department of Experimental and Clinical Medicine, University of Florence, 50121 Florence, Italyrebeccapanconesi@gmail.com (R.P.);; 3 Department of Surgery, A.O.U. Città della Salute e della Scienza di Torino, University of Turin, 10124 Turin, Italy; 4 Liver Unit, Hospital Israelita Albert Einstein, Sao Paulo 05652-900, Brazil; 5 Department of General Surgery and Transplantation, ASST Grande Ospedale Metropolitano Niguarda, 20162 Milan, Italy; 6 Department of Clinical and Experimental Sciences, University of Padua, 35122 Padua, Italy; 7 Department of Surgery and Transplantation, Swiss HPB Center, University Hospital Zurich, 8091 Zurich, Switzerland; 8 Fondazione IRCCS Ca’ Granda, Ospedale Maggiore Policlinico, Center of Preclinical Research, 20122 Milan, Italy; 9 Department of Pathophysiology and Transplantation, Università degli Studi Milan, 20122 Milan, Italy; 10 Transplantation Center, Digestive Disease and Surgery Institute, Department of Immunity and Inflammation, Lerner Research Institute, Cleveland Clinic, Cleveland, OH 44106, USA

**Keywords:** liver transplantation, organ perfusion, dynamic organ preservation, survey

## Abstract

Background: Organ perfusion technology is increasingly used in many countries, with a focus, however, on the Western world. This study investigates the current international trends and obstacles to the broader routine implementation of dynamic perfusion concepts in liver transplantation. Methods: A web-based anonymous survey was launched in 2021. Experts of all involved specializations from 70 centers in 34 countries were contacted, based on the published literature and experience in the field of abdominal organ perfusion. Results: Overall, 143 participants from 23 countries completed the survey. Most respondents were male (67.8%) and transplant surgeons (64.3%) working at university hospitals (67.9%). The majority had experience with organ perfusion (82%), applying mainly hypothermic machine perfusion (HMP; 38%) and other concepts. While most (94.4%) expect a higher utilization of marginal organs with machine perfusion, the majority considers HMP the best technique to reduce liver discard-rates. While most respondents (90%) believed machine perfusion should be fully commissioned, the lack of funding (34%) and knowledge (16%) as well as limited staff (19%) were the three main obstacles to a routine clinical implementation. Conclusion: Although dynamic preservation concepts are increasingly used in clinical practice, significant challenges remain. Specific financial pathways, uniform regulations, and tight collaborations among involved experts are needed to achieve wider global clinical use.

## 1. Introduction

Dynamic organ preservation is an old strategy to improve organs before implantation. Despite the early use in clinical transplantation in the 1970s, only a few high-level clinical studies are available, and technical development is lacking behind medical devices in other healthcare areas [1,2,3,4,5,6]. The clinical application remains, therefore, highly variable and depends on the individual experience and national or center regulations. Quite a few centers worldwide have developed their own perfusion devices, which are, however, frequently not approved by regulatory bodies. One consequence of this non-centralized technology development is the heterogeneous perfusion conditions seen with each perfusion approach and the different devices. Two main perfusion concepts are currently being explored. First, normothermic techniques are performed either in the donor hospital, e.g., as normothermic regional perfusion (NRP) before cold storage and transport, or endischemic thereafter. With the replacement of cold storage during transport (and in the recipient center), normothermic machine perfusion (NMP) was found to reduce liver injury, as shown with less post-transplant liver enzyme release and lower rates of early allograft dysfunction (EAD) [1,7]. The second and different strategy includes hypothermic machine perfusion (HMP) concepts primarily conducted in the recipient center after cold storage and liver transport. This technique was first reported in clinical liver transplantation by Guarrera et al., in 2010 [8]. Following a gap in technological development with a scarcity of good quality studies, the number of randomized controlled trials (RCTs) has only recently increased since 2018, with currently five large RCT cohorts on the role of NMP and HMP [1,2,3,4,5].

Most available data therefore represent, a mix of retrospective cohort studies with individualism and a lack of larger, collaborative approaches and longer-term follow-up. In addition, there is an inherent mismatch between available devices and clinical needs due to a lack of stakeholder partnership. Engineers are rarely seen in operating theatres to determine the clinical requirements, assess the benefits or challenges with specific equipment, or evaluate areas of improvement. Finally, organ transplantation is available mainly in universities and larger hospitals, where the required resources are available to develop and test such new technologies. 

Based on these concerns, this cross-sectional study was designed to explore the current trends and hurdles for a broader clinical application of dynamic perfusion technology in liver transplantation. In addition, a specific focus was placed on obstacles and the need for potential financial concepts to achieve a more regular global use of this technology in the near future. 

## 2. Materials and Methods

### 2.1. Study Design

A web-based questionnaire (https://www.surveymonkey.com/r/XD6ZSTG, accessed on 28 January 2021) was designed using an online platform and launched between January and April 2021. This international survey was also advertised by the International Liver Transplantation Society (ILTS), the International Society of Liver Surgeons (ISLS), and social media (Twitter, Inc.). An overall number of 42 questions was framed, including demographic information, the expectations from machine perfusion technology, and obstacles in implementing this technology in clinical practice (Table 1). The estimated time needed to complete the survey was ≤10 min. The collected data are of social origin and were obtained anonymously. Therefore, ethical approval by an Institutional Board was not deemed necessary.

### 2.2. Study Target

The entire spectrum of staff involved in clinical liver transplantation was invited to participate. Following the initial contact, such experts were asked to circulate the survey link among colleagues in their institution, center, and region. The following participants were actively addressed: transplant surgeons, transplant physicians, anesthetists and intensivists, transplant coordinators, theatre nurses, and industry comprising companies that sell transplant products (e.g., perfusion solutions) or perfusion devices. An overall number of 70 institutions in 34 countries was approached. Experts were approached directly based on the literature published during the past 10 years in the field, which was exceptionally performed by transplant surgeons worldwide. In addition to this strategy, ILTS and ISLS were contacted to advertise the survey on social media to achieve a more representative sample size. The majority of clinicians were based in university hospitals or city hospitals with an active transplant program worldwide. Regarding industry, the employees of five known device companies were approached for participation. Additional staff, namely nurses and coordinators, linked to and accessible through the chairperson or team leader of such departments, were also approached. 

### 2.3. Data Collection

Following survey closure, the data were extracted from the online platform, and a systematic analysis was performed (AP, MFC, AS). The questions addressed the entire spectrum of relevant parameters for liver perfusion, including: demographics (e.g., center size, number, and type of transplants, number of declined organs per year, experience with perfusion), the clinical activity of machine perfusion, expectations from this technology, and the potential benefit regarding specific post-transplant complications, challenges to implement perfusion technology in the participants’ centers and an acceptable price for the required equipment, as well as financial schemes for reimbursement. The detailed questionnaire is attached as Supplementary Table S1.

### 2.4. Data Analysis

Both qualitative and quantitative data were analyzed. The collected data were extracted from the survey platform. For questions where more than one answer could be selected, the answers were regrouped and recalculated. Continuous values were presented as median and IQR, and dichotomic values as number and percent. The chi squared test was used to compare answers provided by different groups, including participants from university hospitals compared to others or responses from participants in centers in Europe/USA/Australia compared to those working in other countries. The demographic data are presented descriptively to detail the sample cohort, with different characteristics, including experience in the field, country of work, position, etc. Based on the clinical experience, the collected data regarding the knowledge and expectations from machine perfusion and the limitations and costs were explored in the context of the participants’ hospital type. Additional subgroup-analysis was performed with a focus on different regions worldwide, comparing North America, Europe, and other countries. The different response rate, also based on the various distribution of organ perfusion technology in countries in North America, Europe, and outside was one reason for this grouping. The analysis was carried out using GraphPad prism Version 7.0. A *p*-value of <0.05 was considered statistically significant.

## 3. Results

### 3.1. Demographical Data

A total of 143 responses were obtained from 50 centers in 23 countries. A total of 136 (95.1%) responses was collected through direct contact using the web link of the survey. An additional seven (5.9%) responses were obtained through the advertisement of the study on social media. Of note, none of the questions was skipped by the participants (answer rate: 100%). The median time to complete the survey was 10 min (IQR: 7–15.5 min). Respondents were mainly male (67.8%; *n* = 97). Leading countries in the field of transplantation were dominantly represented, with 109 participants from Europe/US/Australia (76.2%) and 34 participants (23.8%) from other countries. More specifically, 20.3% responders were from the United Kingdom (UK), 12.6% from Italy and Brazil and 10.5% from the United States of America (USA). Mexico and Switzerland provided 4.2%, and other participating countries were mainly found in Europe, with Portugal and Spain (6.3% each), France, Belgium, Austria, and the Netherlands with 2–3% each. A few responses were also collected from Poland, Denmark, Norway, India, Australia, Chile, and Japan.

Overall, 30.1% were department heads or team leaders, followed by 41.3% consultants and fellows/registrars (5.6% each). Correspondingly, many participants had a vast experience in the field with >20 years (36%), >10 years, and 5 years (34% and 22%) of clinical activity, respectively. Notably, 7.7% of the answers were obtained from transplant nurses and coordinators and 9.1% from colleagues in the related industry. The majority of respondents were transplant surgeons (64.3%). More than 80% worked in university hospitals or large city hospitals (84.7%, *n* = 121), with 47.6% being large volume centers with a total number of ≥100 annual transplant procedures (Table 1). 

Most centers had an active transplant program utilizing donors after circulatory death (DCD; 65.0%, *n* = 93). This picture changed when looking at different regions. Expectedly, centers in countries outside Europe or the USA were more likely not to have a DCD transplant program (*n* = 27, 75.0%). More than half of all respondents (53.1%, *n* = 76) claimed to decline more than 10–30% of organs offered to their center. 

### 3.2. What Are the Experience and Current Clinical Applications of Perfusion Technology?

**Figure 1:**
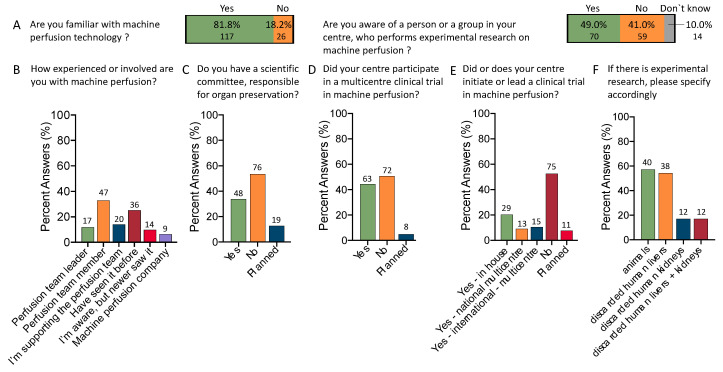
Clinical and scientific activity with machine perfusion: Most respondents were aware of machine perfusion technology (**A**), and about half of the centers had experimental research units (**B**). The majority of participants served as perfusion team leaders or supported the team (**C**). At the same time, most centers did not have a scientific committee leading organ preservation (**D**) and about half of the centers participated in a multicenter clinical trial on the role of machine perfusion (**E**). One-third of centers led or initiated a clinical trial either in-house, nationally, or internationally (**E**). Centers with experimental research focused mainly on studies with animals or discarded human livers; included only 70 participants in a center with experimental research on machine perfusion; centers can have both animals and discarded human organs (**F**).

Most respondents had experience with perfusion technology (81.8% vs. 18.2%), with 12% and 32.3% being either the perfusion team leader or actively involved. An additional 14% provided active support to the perfusion team. Among all participants, 22% had seen or participated in organ perfusion before (Figure 1A,B). Of note, the number of clinicians familiar with machine perfusion was lower in countries outside the USA, Europe, or Australia. Only 20 participants (54%) from such countries, including Mexico, Brazil, and India, had experiences with this technology, compared to 81.8% in the “Western world”. 

Almost half of the participant centers (46.9%) have an active scientific committee (or planned) responsible for the centers’ organ preservation strategy and the defining of future directions (Figure 1C). 

Many participants work in centers with present or previous participation in a clinical trial with organ perfusion (44%, *n* = 63); participation is planned in eight centers (5.6%), in contrast to 50.3% (*n* = 72) without a clinical trial. Notably, only four responders who participated in clinical trials worked outside Europe/US/Australia. More specifically, 39.9% of survey respondents work in centers which led a clinical trial, either in-house (*n* = 29), nationally (*n* = 13) or internationally (*n* = 15) (Figure 1D). In addition, half of all responders (49%, *n* = 70) have a research team or unit where organ perfusion is tested in experimental studies, mainly using animal livers (*n* = 40/70, 57.1%) or discarded human livers (*n* = 38/70, 52.3%) (Figure 1E,F). 

**Figure 2:**
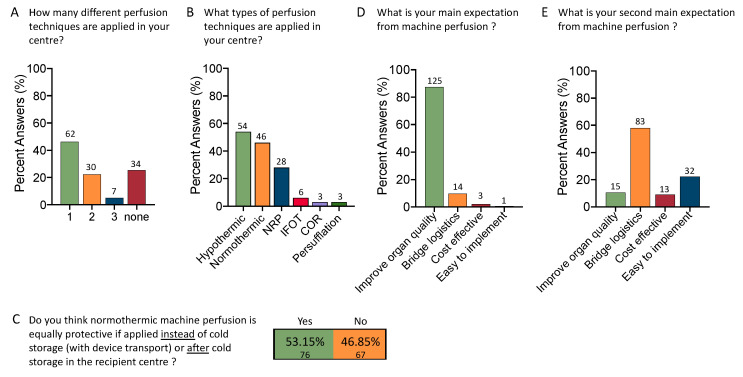
Current clinical use of machine perfusion technology and related expectations: Most centers use organ perfusion in clinical practice, and many do or have tested more than one technology (**A**); With >50%, most centers used some sort of hypothermic perfusion technique, followed by normothermic machine perfusion (NMP) and normothermic regional perfusion (NRP) (**B**); Centers that use perfusion technology in general (34 without excluded); 37 centers employ more than one different technique. Despite several publications on an equally high rate of ischemic cholangiopathies with endischemic NMP, more than half of the participants still believe that NMP is equally protective when conducted at the recipient center after a relevant period of cold storage (53.2%) (**C**). Therefore, the first main expectation is that dynamic perfusion techniques improve organ quality (87.4%), followed by support with logistical challenges (9.8%) (**D**). Interestingly, the need for support with logistical issues was listed by most participants as the second main expectation (58.0%), followed by an easy implementation (22.4%) (**E**).

Given the increasing number of perfusion devices with three main perfusion strategies, it appeared interesting that the majority used or tested only one approach (*n* = 62, 46%); two or three different perfusion techniques were explored in 22% (*n* = 30) and 5% (*n* = 7) of participating centers, respectively (Figure 2A). The most commonly used technologies were ex situ HMP (38.6%), followed by normothermic machine perfusion (NMP, 32.9%) and in situ normothermic regional perfusion (NRP; 20%). Other techniques, e.g., “ischemia-free” organ transplantation (IFOT), controlled oxygenated rewarming (COR), and organ persufflation, were applied with 4%, 2%, and 2%, respectively (Figure 2B). Interesting was also the topic of the best modality of NMP. Despite available literature [9,10], most respondents believe that NMP achieves similar post-transplant outcomes when performed as a back-to-base approach in the recipient center (53.2%, *n* = 76) compared to upfront NMP starting at the donor center (Figure 2C).

Next, the field expects an organ quality improvement with perfusion technology (87.4%, *n* = 125). Providing the same answer options again to capture the second main expectation, most participants selected the need for support with logistical challenges (58.0%, *n* = 83) or an easy-to-implement technology (22.4%). Nine percent selected cost-effectiveness as a key parameter (Figure 2D,E).

### 3.3. What Is the Role of Perfusion Technology in the Utilization of Marginal Organs?

**Figure 3:**
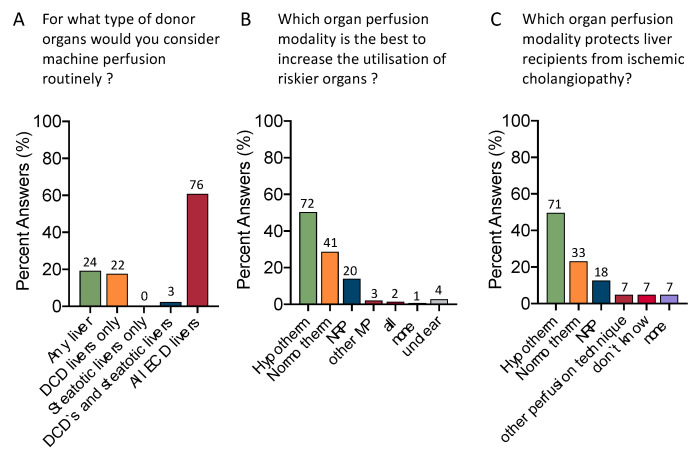
Current clinical application and effect on marginal organs: Of interest were results regarding clinical use and the effect of organ perfusion techniques. The vast majority of respondents selected hypothermic perfusion techniques as the most promising approach to increase utilization of risky organs (**A**); other MP: *n* = 3 (1× COR, 1× IFOT, 1× NRP combined with other perfusion techniques). Most participants would perfuse all types of extended criteria donor organs and of interest is that 20% would perfuse any liver before transplantation (**B**). The majority would expect hypothermic machine perfusion to protect liver recipients from ischemic cholangiopathy (50%), followed by NMP (23%) and NRP (12.6%) (**C**); other perfusion techniques included 4× NRP combined with HOPE, 1× IFOT; 2 respondents considered available data insufficient to provide an answer.

The next interesting topic is the impact of perfusion technology on donor utilization. To improve organ quality, the majority (60.0%, *n* = 76) would perfuse all types of extended criteria donor (ECD) livers (Figure 3A). Of note, 19.2% would perfuse any liver (*n* = 24).

Half of all participants (*n* = 72, 50.3%) would consider HMP as the best technique to increase the utilization of riskier grafts, followed by NMP (*n* = 41, 28.6%) and NRP (*n* = 20, 14%) (Figure 3B). The vast majority (49.7%, *n* = 71) selected HMP as the best tool to protect recipients from ischemic cholangiopathy (IC), followed by NMP (23.1%, *n* = 33) and NRP (12.6%, *n* = 18) (Figure 3C). 

**Figure 4:**
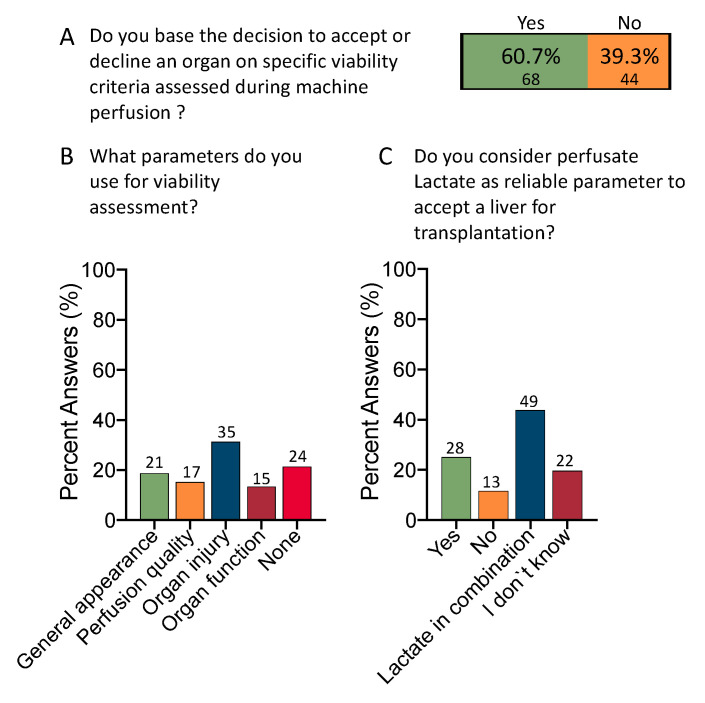
Current role of viability testing. The majority of centers with active perfusion programs use some sort of viability tests for organ assessment (60.7% vs. 39.3% (**A**), respondents included with active machine perfusion programs (*n* = 112). Most colleagues use macroscopic parameters, markers of perfusion quality or organ function at almost equal percentages (18.8%, 15.2%, and 13.4%), while slightly more respondents base their organ assessment on parameters of organ injury (31.3%) (**B**); 34 participants were excluded because their center did not perform organ perfusion or participant worked for a company. One quarter (25%) would still consider lactate a reliable marker of viability assessment. Most participants would, however, combine lactate with other viability markers (43.8%) (**C**). IFOT: ischemia free organ transplantation; COR: controlled oxygenated rewarming; NMP: normothermic machine perfusion; NRP: normothermic regional perfusion.

Most participants would base their decision to accept an organ on some sort of viability assessment during organ perfusion (60.7%, *n* = 68) (Figure 4A). Although markers of organ injury were selected as the leading group of viability tests (31.3%, *n* = 35), there are heterogeneity and organ parameters (18.8%, *n* = 21), perfusion parameters (15.2%, *n* = 17), and markers of organ function (13.4%, *n* = 15), considered almost equally. In addition, most clinicians combine lactate with other markers (43.8%, *n* = 49) (Figure 4B,C).

### 3.4. What Are the Current Challenges in Implementing Perfusion Technology in Routine Clinical Practice?

**Figure 5:**
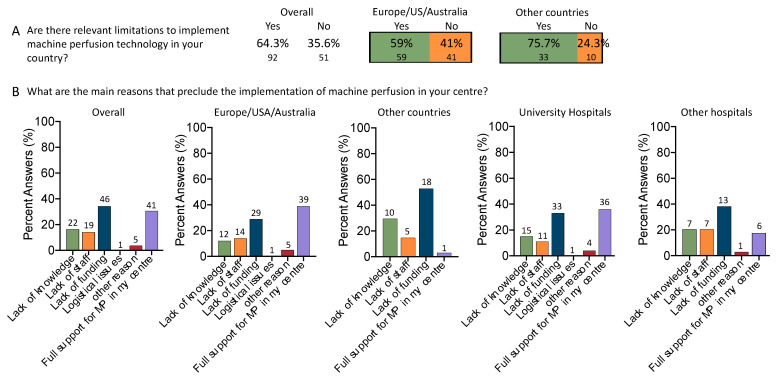
Hurdles and limitations to implementing perfusion technology in clinical settings: In the majority of centers, relevant hurdles to implementing perfusion technology exist (64.3%), however, at a higher percentage in countries outside the Western world (75.7% in countries other than Europe/USA/Australia; 59% in centers from the Western world) (**A**). Lack of funding (34.3%), lack of knowledge (16.4%), and lack of staff (14.2%) were the three main reasons listed by many working with liver transplantation. Interestingly, almost no participant prioritized logistical issues as the cause of the delayed implementation of this technology. Of note, funding gains were of even more importance when assessing countries outside the Western world. More than half of the centers (52.9%) listed the lack of funding as the main reason, compared to 29% in centers from Europe/USA/Australia. Even university hospitals claim that funding is the main issue (33%), which was similar compared to other smaller hospitals (38.2%). Such responses were compared using the chi squared test (level of significance comparing Europe/USA/Australia vs. other hospitals: *p* < 0.001 and university hospitals vs. other hospitals: *p* = 0.166) (**B**).

Almost two thirds of survey participants (64.3%, *n* = 92) admit that there are significant hurdles in implementing this technology in their clinical practice (Figure 5A). The proportion of centers experiencing such challenges was higher in countries outside Europe, US, or Australia. With a higher number of participants from centers and university hospitals located in Europe, US, or Australia (59/143, 41.2%), the overall number of centers with hurdles to implement organ perfusion technologies was higher here, as opposed to other countries (33/143, 23% related to the overall cohort). The majority (overall cohort) selected lack of funding as their primary obstacle, with 34.3% (*n* = 46), followed by lack of knowledge (16.4%, *n* = 22), and limited availability of staff (14.2%, *n* = 19). Only one-third (*n* = 41/134, 30.6%) of participants stated full support in their center in promoting this technology (Figure 5B). As expected, such findings changed in accordance with the participants’ region or country in the world. The financial support and knowledge of this technology are much lower in countries outside Europe or the USA. More than 50% selected lack of funding as the main challenge (51.4%, *n* = 18). Similarly, the lack of knowledge and staff limitation were higher (29.4%, *n* = 10; 14.7%, *n* = 5) in such countries. This was further paralleled by a decline in participants working in centers with full support for implementing this new technology (from 39.0% to 2.9% in other countries) (Figure 5B). Interestingly, the lack of knowledge is also listed as the second main limitation in universities and larger city hospitals (15% vs. 20%). 

As expected, the lack of staff appears also higher in non-university hospitals. Of note, transplant units in university hospitals are more frequently fully supported with novelties than larger city units.

### 3.5. What Is an Acceptable Price for Organ Perfusion Technology?

**Figure 6:**
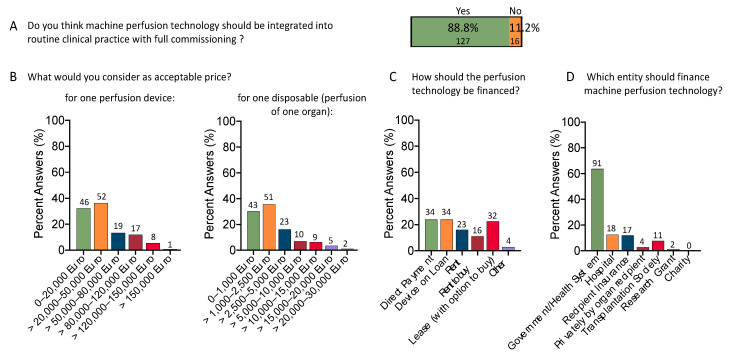
Costs and finances of organ perfusion technology: Almost 90% of all participants support the integration of organ perfusion technology in routine practice with full commissioning (88.8% vs. 11.2%) (**A**). A price of up to EUR 50,000 was nominated as acceptable for one perfusion device by most (68.5%). Still, only the minority of 6.3% would pay >120,000 EUR, reflecting the financial capabilities of most transplant centers worldwide. A similar picture was seen with the costs for one single perfusion, e.g., the price for one disposable. Most participants would consider up to EUR 2500 acceptable for one organ perfusion (**B**). Based on the different environment of study participants, the financial schemes suggested for this technology vary considerably. Direct device payment, device on loan or lease with option to buy were selected by a comparable number of participants (23.8%, 23.8%, 22.4%), followed by rent (16.1%) and rent to buy (11.2%) (**C**); Other financial schemes were listed as: *n* = 2 purchase by OPO and *n* = 1 as I don’t know, with negotiations. The majority selected government/health systems to reimburse hospitals for equipment costs (63.6% (**D**).

With an increasing number of devices on the market, it becomes more evident how costly this technology appears compared to medical devices in other healthcare sectors. Table 2 describes the current device landscape for ex situ machine liver perfusion. While most survey participants (88.8%, *n* = 127) consider this technology efficient and worthy of full commissioning, costs and effects should be balanced (Figure 6A). The majority of responders considered a device price of up to EUR 50,000 as reasonable (68.5%, *n* = 98). Notably, 21 (21.4%) of these responders were from countries outside Europe/US/Australia. Only five participants (3.4%) from Europe/US/Australia and four participants (2.7%) from other countries would pay more than EUR 120,000. Such results are further paralleled by the accepted costs for one disposable, e.g., one individual organ perfusion, frequently while not even considering additional costs for solutions and other components. Two-thirds of the participants selected a price up to EUR 2500 acceptable for a single disposable (65.7%, *n* = 94), of whom 73 were from Europe/US/Australia and 21 from other countries. A few colleagues would pay between EUR 2500 and EUR 5000 (16.1%, *n* = 23) (Figure 6B). Discriminating between experts working in institutions of different sizes led to the same results (Supplementary Figures S1 and S2). 

Based on the different environment, countries, and social security systems of the study participants, the financial schemes suggested to compensate for this technology vary considerably. Direct device payment, device on loan, or lease with option to buy were selected by a comparable number of participants (23.8%, *n* = 34; 23.8%, *n* = 34; 22.4%, *n* = 32), followed by rent (16.1%, *n* = 23) and rent to buy (11.2%, *n* = 16). The majority selected government/health systems to reimburse hospitals for the equipment costs (63.6%, *n* = 91), followed by hospitals, recipient insurance, or transplant societies (Figure 6C,D). 

## 4. Discussion

This international survey paints an interesting picture of the current clinical use of perfusion technology for liver transplantation and highlights relevant obstacles that delay efficient implementation in routine clinical practice. The study showed the following main results. First, most participants were consultant surgeons and perfusion team leaders or members working in large-volume transplant centers at university hospitals in 26 countries worldwide. With the overall expectation to improve donor organ quality, most participants nominated HMP concepts as the best possible tool to increase marginal organ utilization and prevent IC after liver transplantation. The second most relevant expectations were the need to “bridge logistical challenges” and the requirement for the technology to be easy to implement in the already challenging environment of organ donation and transplantation. Finally, the lack of financial support was the dominant cause of an overall slow integration into routine practice, described equally by participants from universities and “smaller hospitals” and found even more pronounced in countries outside Europe and USA.

While the vast majority believe that dynamic preservation concepts are effective enough to be commissioned by the government or national health systems, equipment prices should balance the effect. Despite major differences in how healthcare costs are reimbursed worldwide, most study participants would limit the device and disposable expenses to a maximum of EUR 50,000 and EUR 2500, respectively. 

Organs are declined for various reasons. Donor livers can be rejected upfront due to a presumed too high risk or based on organ factors evident at procurement surgery or thereafter [11]. In our complex transplant system, logistical challenges, including the lack of specialized theatre, intensive care staff, or a limited bed capacity, are of additional relevance to safely accept an organ and avoid too long a cold ischemia time [12]; not forgetting recipient risk factors, which impact overall outcomes even after the implantation of a liver, that previously met all viability criteria obtained during perfusion [13]. Most clinicians expect a higher utilization of risky organs and would apply perfusion techniques in organs from ECD, including DCDs and steatotic grafts. A good example is Sweden, where an expert committee currently defines national criteria for DBD–ECD donor livers to undergo routine hypothermic oxygenated perfusion (HOPE), considering available criteria for marginal liver grafts [11]. Similar approaches towards commissioning this technique are seen in other countries, including the Netherlands and Belgium, based on the increasing number of RCTs available. Clinical studies demonstrating an impact of perfusion not only on early allograft dysfunction but on clinically relevant post-transplant outcomes, such as major complications and improved graft survival rates, are the main drivers for such changes [2,3,4]. Other countries, including Italy and Switzerland, routinely use NRP and HOPE techniques, with both approaches commissioned for DCD liver transplants [14,15]. The situation is similar in Spain, France, and parts of the UK, where abdominal type-III DCD donor organs are routinely procured with NRP7 [16,17]. 

As organ donation rates grow in many countries and more indications are accepted for transplantation, overall transplant numbers are likely to rise. However, the transplant profession appears to be a less popular career for future generations due to the physical and mental demands [18,19]. Furthermore, given the trajectory of donor demographics with more organs coming from elderly donors with multiple co-morbidities, the stress of decision-making to increase organ utilization will likely worsen the current situation. Recently, an international expert meeting in liver perfusion was held in Turin [20]. Experts illustrated the current knowledge of organ perfusion technology and ongoing clinical and experimental studies. The results of the survey displayed here, obtained from 26 countries worldwide, are further paralleled by the overall findings presented by such experts during the Turin meeting. The present survey provided additional information on the current real-life challenges related to liver utilization in different countries, the obstacles to progress and routine use. 

Despite the need for revenue for perfusion companies to achieve new investments and push for the development of better devices in the future, current economic analyses in the UK classify perfusion technology as financially not sustainable with a low probability of achieving cost-efficiency [21]. In times of limited resources and the need for cost-benefit, the widespread use of perfusion devices could be hampered by their additional costs. As expected, our study showed that the main obstacle to implementing this technology was the lack of financial support. Currently, NMP techniques are more complex compared to hypothermic approaches, increasing the resources required to perform the technique. In addition, costs depend on factors such as device transport, perfusate composition with the need for red blood cell concentrates and other additives, required staff, the countries tax rates, and distributor margins [22]. Due to its complexity, most devices for NMP are supplied on loan or with a service approach. While disposable costs for one perfusion were historically supplied at lower prices for devices capable of hypothermic and normothermic perfusion, such companies have recently increased their prices. The cost for one liver NMP ranges, therefore, today between EUR 10,000–100,000. In contrast, the use of NRP for DCD procurements is commissioned in a few countries in Europe with an average price between EUR 2500 and 3500. Equipment needed for one hypothermic perfusion is also significantly cheaper compared to NMP technology, and ranges between EUR 3500 and 12,000 for most devices. Hypothermic techniques are also less labor intensive and require fewer additional components for the perfusion solution. Of note, two-thirds of the study participants considered up to EUR 2500 as an acceptable price for the perfusion of one organ. 

Current prices for other lifesaving healthcare procedures are of interest in this context. For example, a hemodialysis device with disposables may well cost between EUR 8000 and USD 10,000 with annual costs of EUR 87,600 and USD 108,000 per patient [23,24,25,26]. Pacemakers come at an average price between USD 5000 and 10,000. Interestingly, a commonly used pacemaker can cost USD 4200 in the USA and USD 1400 in Germany [27]. Such healthcare equipment has been on the market for many decades, which might well be one reason for the lower costs seen today compared to organ perfusion technology. The clinically most effective perfusion technique, provided by a company with affordable and easy-to-use equipment that overcomes additional logistical burdens and provides reliable viability testing, will potentially win most of the market in the future. 

In addition, higher utilization of DCD livers would be expected. It has been shown that DCD liver transplantation has 25% to 30% higher costs than livers from donors after brain death, mainly due to the increased need for interventions, expensive antibiotics, and re-transplantation based on a higher incidence of biliary complications [28]. In this setting, given the results from multicenter randomized controlled trials [2,3,4], the costs of HOPE could be justified by better outcomes. For example, a trial from Germany found shorter hospital stays and fewer complications within the first three months. A cost analysis demonstrated the cost-benefit of the HOPE concept [29]. This was further paralleled by a recent study from France, where HOPE treatment did not lead to higher costs despite the additional need of EUR 5298 per patient [30]. However, most cost studies lack specific key contributors to paint the overall picture. Either the costs for organ donation or post-transplant complications, or the benefit of utilizing more organs is lacking. Reducing candidates waiting time for a liver with fewer treatments on the waiting list (e.g., trans-arterial chemo-embolization for hepatocellular carcinoma candidates, antibiotics) will amortize certain perfusion equipment costs and should be considered for such analyses. Good quality cost studies are not yet available. With an increasing understanding of how relevant the reduction of post-transplant complications is and the achievement of better graft survivals, such studies are well expected in the near future [4]. Recently, Patrono et al. [31] performed a survey to evaluate the utilization of MP in liver transplantation. The authors proposed 10 clinical scenarios, enquiring the responders about organ utilization and what type of MP they would use. The results paralleled the findings in our study here. 

The majority of respondents preferred HMP and an end-ischemic perfusion approach in 56.3% and 81.1% of cases, respectively. Although this study parallels our findings, Patrono et al., did not evaluate the obstacles in implementing MP in current clinical practice and their study focused more on liver viability assessment. Above all, their study included responders from Europe. In contrast, we targeted well-known liver transplant centers in 23 countries worldwide, providing a better global picture of the current hurdles to increase MP utilization, despite improved clinical outcomes.

Many transplant centers have currently limited access to perfusion devices, with an impact by local or regional financial constraints [32]. In the US, it is estimated that machine perfusion could at least add USD 25,000–50,000 to the cost of a single liver transplant [32]. This takes into consideration the expenditures of the device, disposables, required staff, blood products, pharmaceutical agents, and other costs not incurred with SCS32. The American Society of Transplant Surgeon has suggested that American transplant centers and organ procurement organizations (OPOs) should develop a mutually agreeable approach to perfusion in their specific donation service area [33]. In addition, companies are required to tailor their approach in different regions worldwide to the available resources of the local healthcare system and related reimbursement structures, as routinely done by experienced companies with medical devices other than for organ perfusion. Expensive devices are on loan for a specific period with the commitment to a certain number of procedures, where the consumable price includes the device loan. Interestingly, many respondents of this survey would be in favor of this financial model or similar. In contrast, countries such as India, where the liver recipient finances the transplant procedure, may require a different approach. Experienced centers with financial support may pay for the device directly to enable flexible cost reduction for each consumable in collaboration with the device company and distributors. 

Despite such interesting findings, our study has certain limitations. First, the number of participants depends on the provided access through expert societies, social media, and the interest of the target. Surgical chairs in transplantation may have distributed the survey link to their team; however, there were limited control mechanisms for completing the survey. This could lead to a selection bias. While our study covered the surgical teams and perfusion group leaders, who provide direction regarding applied organ preservation in practical terms, other caregivers, e.g., anesthetists, nurses, and coordinators, are underrepresented. More than 80% of responders had experience with perfusion technologies, highlighting the fact that the survey was completed by persons with certain expertise in the field, thus allowing a selection bias. Smaller and mid-volume centers, that are currently starting or planning to do organ perfusion are largely not yet connected to the ever-growing collaborative networks of liver perfusion and were therefore not captured. An additional study on such smaller and mid-volume centers would therefore be of interest to discuss their specific challenges. Next, the unawareness of the purpose of the investigation or the lack of knowledge of this technology might have led to limited participation in some of these cohorts. This might have led to the relatively high number of surgeons among participants and their primary localization in university hospitals. The results should also be interpreted with caution regarding the participant’s country. Transplant units are unavailable in some countries worldwide, and some regions and continents, such as Africa, are under-represented. The type of transplant program also affects perfusion technology; Asian participants are consequently low in number due to the dominance of living donor liver transplantation. Some findings could have been even more pronounced when the survey was applied in more rural areas or focused on different cohorts. The time point when the survey was carried might also have an impact due to the lack of important studies available in the meantime. Finally, as the technology and the entire field appears very dynamic and the implementation of this technology is currently in the process of increasing, this study is only a first “snapshot” of the current situation; a repeat performance of the survey in a few years could be of interest to the development in the field. 

In summary, this international survey showed an increasing use and knowledge of organ perfusion technology in many countries with active deceased liver transplant programs to improve donor utilization at higher safety through better organ quality. Most experts believe dynamic preservation concepts should be commissioned for routine use at lower prices than currently available. Indeed, the lack of financial support is the leading obstacle to broader implementation. However, with more studies showing an impact on post-transplant complications and graft survival with a clear cost-benefit, commissioning could be achieved in an increasing number of countries.

## Figures and Tables

**Table 1:** Overview of demographic survey results.

Parameter	Characteristics		Parameter	Characteristics	
Employment Position	Department Head: Consultant: Registrar: Fellow: Nurse: Other (Industry, coordinator):	30.1% (*n* = 43) 41.3% (*n* = 59) 5.6% (*n* = 8) 5.6% (*n* = 8) 7.7% (*n* = 11) 9.8% (*n* = 14)	Number of Transplant Procedures annually per center	>300: 201–300: 151–200: 101–150: 51–100: 21–50: 0– 20: n.a.	7.0% (*n* = 10) 14.7% (*n* = 21) 12.6% (*n* = 18) 13.3% (*n* = 19) 32.2% (*n* = 46) 10.5% (*n* = 15) 4.9% (*n* = 7) 4.9% (*n* = 7)
Working Role	Transplant Surgeon: Physician: Transplant coordinator: Intensive Care Nurse: Anesthetist/Intensivist: Theatre Nurse: Other (Industry):	64.3% (*n* = 92) 15.4% (*n* = 22) 4.9% (*n* = 7) 3.5% (*n* = 5) 1.4% (*n* = 2) 1.4% (*n* = 2) 9.1% (*n* = 13)	DCD transplant program	Yes No Not applicable §	65.0% (*n* = 93) 28.7% (*n* = 41) 6.3% (*n* = 9)
Gender	Female: Male:	32.2% (*n* = 46) 67.8% (*n* = 97)	Declined livers annually *	> 30% 21–30% 11–20% 6–10% 0–5% Don’t know	8.8% (*n* = 11) 16.0% (*n* = 20) 32.0% (*n* = 40) 28.0% (*n* = 35) 8.0% (*n* = 10) 7.2% (*n* = 9)
Age	36–45 years: 46–55 years: 56–65 years: 26–35 years: >65 years:	44.1% (*n* = 63) 23.1% (*n* = 33) 16.1% (*n* = 23) 12.6% (*n* = 18) 4.2% (*n* = 6)	Region	Europe: USA: Other (e.g., Central and South America, Asia, Australia)	64.3% (*n* = 92) 10.5% (*n* = 15) 25.2% (*n* = 36)
Working Experience (years)	>20 years: >10–20 years: >5–10 years: >2–5 years:	35.7% (*n* = 51) 33.6% (*n* = 48) 21.7% (*n* = 31) 9.1% (*n* = 13)	Hospital type and Working environment	University Hospital: City/Regional Hospital: Covering regions and industry: Other types of Hospital:	67.9% (*n* = 97) 16.8% (*n* = 24) 6.3% (*n* = 9) 2.8% (*n* = 4)

§ Participants working for a device company; * Represents the number of declined organs annually.

**Table 2:** Overview of the current landscape of machine perfusion technology used for ex situ perfusion in clinical studies or routine practice.

Device Name	Company and Country of Production	Perfusion Modality	Organs	First Clinical Use	Leading Countries of Use	Disposable Costs	Achieved Approvals	Ongoing Prospective Trials and RCTs
Metra^®^	Organox, United Kingdom	Normothermic Machine Perfusion	Liver	2013, UK	UK, Austria, Germany	$$$	CE mark, FDA approved	USA, Germany, Spain
OCS^TM^ liver^®^	Transmedics, USA	Normothermic Machine Perfusion	Liver	2016, UK and USA	USA	$$$$	CE mark, FDA approved	None
Liver Assist^®^	XVIVO (Organ Assist), Sweden (The Netherlands)	Normothermic and Hypothermic Oxygenated Perfusion	Liver	2012, Switzerland	The Netherlands, Belgium, Italy, France, Sweden	$–$$	CE mark	France, Netherlands, Poland, Germany
PerLife^®^	Aferetica, Italy	Normothermic and Hypothermic Oxygenated Perfusion	Liver, kidney	2021, Italy	Italy	$$	CE mark	Italy, Germany
Lifeport liver transporter^®^	Organ Recovery Systems, USA	Hypothermic Machine Perfusion	Liver	2019, USA	USA	$$	FDA approval expected in 2023	USA
VitaSmart^®^	Bridge to life, USA/Europe (production: Medica, Italy)	Hypothermic Oxygenated Perfusion	Liver, kidney	2018, Italy	Switzerland, Portugal, Sweden, India, UK, France	$–$$	CE mark, ongoing RCT for FDA approval	USA, France, Germany, Italy

Costs for one disposable are presented in categories: $: ≤ 10,000; §§ >10,000 to 30,000; $$$: >30,000 to 50,000 USD; $$$$ > 50,000 USD; Costs for one ex-situ perfusion may also include the perfusion solutions and does vary among countries based on distributor costs, margins, individual payment schemes and tax rates. Disposable costs and ranges are often not available for certain regions and countries and may significantly differ worldwide.

## Data Availability

All data are available upon reasonable request.

## Supplementary Materials

The following supporting information can be downloaded at: https://www.mdpi.com/article/10.3390/jcm12113765/s1, Figure S1: Subgroup analysis on accepted costs for one perfusion device according to hospital type; Figure S2: Subgroup analysis on accepted costs for one organ perfusion (one disposable); Table S1: Survey Questionnaire.
