# Supplementary material for: Trends and Obstacles to Implement Dynamic Perfusion Concepts for Clinical Liver Transplantation: Results from a Global Web-Based Survey
Source: J Clin Med. 2023 May 30;12(11):3765. doi: 10.3390/jcm12113765 (PMC10253826; doi:10.3390/jcm12113765)
Supplement: Supplementary file 1 [file jcm-12-03765-s001.zip › jcm-2346294-supplementary.pdf]

Trends and obstacles to implement dynamic perfusion concepts for clinical liver transplantation: Results from a global web-based survey

Alessandro Parente [1], Mauricio Flores Carvalho [2], Rebecca Panconesi [2,3], Yuri L. Boteon [4], Riccardo De Carlis [5,6], Philipp Dutkowski [7], Paolo Muiesan [2,8], Daniele Dondossola [8,9] and Andrea Schlegel [2,7,8,10,*]

[1] HPB and Transplant Unit, Department of Surgical Science, University of Rome Tor Vergata, 00133 Rome, Italy; aleparen@gmail.com
[2] Department of Experimental and Clinical Medicine, University of Florence, 50121 Florence, Italy; drmauras@gmail.com (M.F.C.); rebeccapanconesi@gmail.com (R.P.); paolo.muiesan@unimi.it (P.M.)
[3] Department of Surgery, A.O.U. Città della Salute e della Scienza di Torino, University of Turin, 10124 Turin, Italy
[4] Liver Unit, Hospital Israelita Albert Einstein, Sao Paulo 05652-900, Brazil; yurimed43@yahoo.com.br
[5] Department of General Surgery and Transplantation, ASST Grande Ospedale Metropolitano Niguarda, 20162 Milan, Italy; riccardo.decarlis@ospedaleniguarda.it
[6] Department of Clinical and Experimental Sciences, University of Padua, 35122 Padua, Italy
[7] Department of Surgery and Transplantation, Swiss HPB Center, University Hospital Zurich, 8091 Zurich, Switzerland; philipp.dutkowski@usz.ch
[8] Fondazione IRCCS Ca' Granda, Ospedale Maggiore Policlinico, Center of Preclinical Research, 20122 Milan, Italy; dondossola.daniele@gmail.com
[9] Department of Pathophysiology and Transplantation, Università degli Studi Milan, 20122 Milan, Italy
[10] Transplantation Center, Digestive Disease and Surgery Institute, Department of Immunity and Inflammation, Lerner Research Institute, Cleveland Clinic, Cleveland, OH 44106, USA
* Correspondence: schlega4@ccf.org

Keywords: liver transplantation, organ perfusion, dynamic organ preservation, survey.

Author contributions:

Study concept: AP, MFC, PM, AS
Survey design: AP, MFC, AS
Data collection: AP, YLB, RDC, PM, AS
Data analysis and interpretation: AP, PD, AS
Manuscript writing: AP, AS
Manuscript revision and approval: all authors
AP and MFC contributed equally as shared first authors

Abbreviations:
COR: controlled oxygenated rewarming; DBD: Donation after Brain Death; DCD: Donation after circulatory death; EAD: Early allograft dysfunction; ECD: Extended Criteria Donor; HMP: Hypothermic Machine Perfusion; HOPE: Hypothermic Oxygenated Perfusion; ICU: Intensive care unit; IFOT: "ischemia free organ transplantation"; MP: Machine perfusion; NRP: Normothermic regional perfusion; NMP: Normothermic Machine Perfusion; PNF: Primary non function;

Layout:

1) Supplementary Figures S1 and S2
2) Supplementary Table S1: Survey questions

1. Supplementary Figures

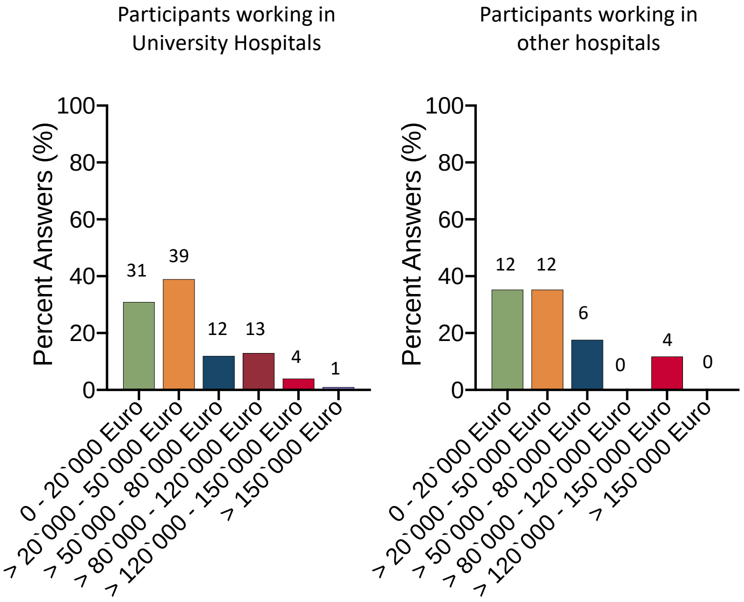

Supplementary Figure S1: Subgroup analysis on accepted costs for one perfusion device according to hospital type.

The templates included only respondents from university hospitals (left) and other hospitals (right), n=100 and n=34, respectively.

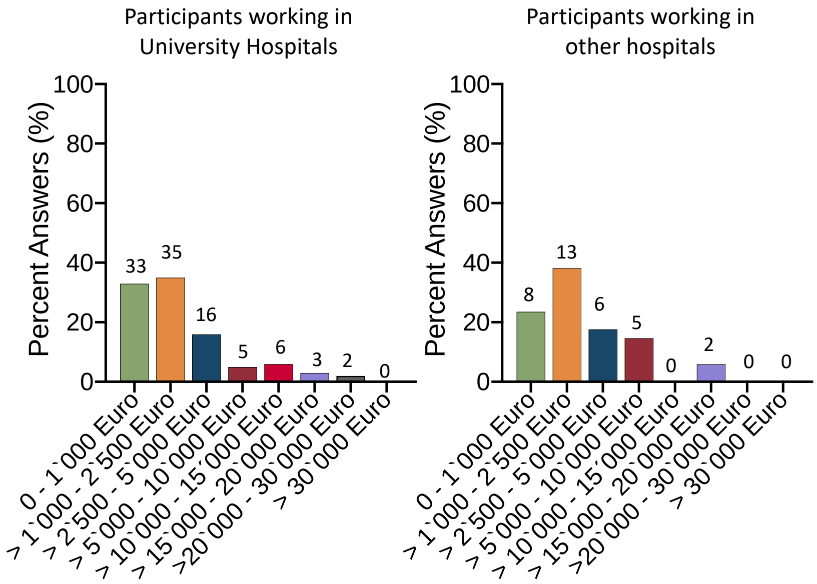

Supplementary Figure S2: Subgroup analysis on accepted costs for one organ perfusion (one disposable).

The templates included only respondents from university hospitals (left) and other hospitals (right), n=100 and n=34, respectively. Ten participants worked for med tech companies, selling equipment for organ perfusion worldwide: 90% (n=9/10) would accept up to 5`000 Euro per one disposable/organ perfusion; 30% (n=3) 0-1`000Euro; 30% (n=3) 1000-2500Euro and 30% (n=3) 2500-5000 Euro. One respondent would pay up to 15`000 Euro.

2. Supplementary Table S1: Survey Questionnaire:

**Machine Perfusion in abdominal organ transplantation**
**Q1. What is your gender?**
**Answer Choices**
Female
Male
Other (specify)
**Q2. In what country do you work?**
select
**Q3. What is your age?**
**Answer Choices**
18-25
26-35
36-45
46-55
56-65
> 65
**Q4. Hospital / Unit**
**Answer Choices**
University Hospital
Large City / regional Hospital

Other Type of Hospital

Covering more than one region (coordinator, industry,...)

Other (please specify type of hospital)

Q5. Staff Role

Answer Choices

Transplant Surgeon

Physician

Anaesthetist

Intensivist

Intensive Care Nurse

Theatre Nurse

Transplant Coordinator - Donor

Transplant Coordinator - Recipient

Other (please specify)

Q6. Employment Position

Answer Choices

Head of Department / Team Leader

Consultant

Registrar

Fellow

Nurse

Other (please specify)

Q7. How many years of clinical experience do you have ?

Answer Choices

> 2 - 5

> 5 - 10

> 10 - 20

> 20

**Q8. How many transplant procedures are done annually in your centre?**
**Answer Choices**
0 - 20
21 - 50
51 - 100
101 - 150
151 - 200
201 - 300
> 300
Not applicable

**Q9. Which organs do you and your team deal with ?**
**Answer Choices, more than one**
Liver
Kidney
Pancreas

**Q10. How many deceased LIVER transplant procedures are done annually in your centre?**
**Answer Choices**
0 - 20
21 - 50
51 - 100
101 - 150
151 - 200
201 - 300
> 300
Not applicable

**Q11. How many deceased KIDNEY transplant procedures are done annually in your centre?**
**Answer Choices**
0 - 20

21 - 50

51 - 100

101 - 150

151 - 200

201 - 300

> 300

Not applicable

Q12. How many pancreas transplantations are done annually in your centre ?

Answer Choices

0 - 5

6 - 10

11 - 15

16 - 20

21 - 50

> 50

Not applicable

Q13. Do you have a donation after circulatory death (DCD) transplant program in your centre ?

Answer Choices

Yes

No

I don`t know

Q14. Are you familiar with machine perfusion technology ?

Answer Choices

Yes

No

Q15. How experienced or involved have you been in machine perfusion ?

Answer Choices

Have seen it before
Have supported the perfusion team before
Actively involved as a member of the perfusion team or study team
I`m aware of this technology, but have never seen it
I`m not aware of machine perfusion technology
Perfusion team leader
I‘m working for a machine perfusion company

**Q16. Do you have a specific clinical/scientific committee in your centre or department, who decides what preservation or perfusion strategy to implement ?**
**Answer Choices**
Yes
No
Planned to be implemented

**Q17. What perfusion techniques or strategies are routinely performed in your centre (please select more than one answer if appropriate) ?**
**Answer Choices**
Normothermic Regional Perfusion (NRP)
Hypothermic Oxygenated Perfusion (HMP, HMP-O2, HOPE, D-HOPE)
Normothermic Machine Perfusion (NMP)
Controlled Oxygenated Rewarming (COR)
Oxygen Persufflation
Ischemia Free Organ Transplantation (IFOT)
None
Other (please specify)

**Q18. Did your centre participate or have you recently participated in a multicentre clinical trial on machine perfusion ?**
**Answer Choices**
Yes

No

Planned

Q19. Did your centre initiate or lead a clinical trial in machine perfusion ?

Answer Choices

Yes - in house

Yes - national multicentre

Yes - international - multicentre

No

Planned

Q20. Are you aware of a person or a group in your centre, who performs experimental research on machine perfusion ?

Answer Choices

Yes - there is experimental research in my centre (laboratory)

No

I don`t know

Q21. If there is experimental research, please specify accordingly.

Answer Choices

Experimental research with animals

Experimental research with discarded human LIVERS

Experimental research with discarded human KIDNEYS

Experimental research with discarded human LIVERS and KIDNEYS

Not applicable

Q22. How many organs are declined in your institution annually? Please select percentage.

Answer Choices

0 - 5 %

6 - 10 %

11 - 20 %

21 - 30 %

> 30 %

Not applicable

Other (please specify)

Q23. What is the main reason to decline a Liver in your centre ?

Answer Choices

Donor risk (e.g. age, medical risk, lab values,...)

Organ quality (macroscopy, perfusion quality during procurement, donor warm ischemia time, ...)

Recipient disease severity (medically unfit for procedure)

Logistical issues (e.g. ITU / Theatre capacity)

NONE-we are not doing liver transplantation in my centre

Not applicable

Other (please specify)

Q24. What is the second main reason to decline a Liver in your centre ? (chose a different answer please)

Answer Choices

Donor risk (e.g. age, medical risk, lab values,...)

Organ quality (e.g. macroscopy, perfusion quality during procurement, donor warm ischemia time,...)

Recipient disease severity (medically unfit for procedure)

Logistical issues (e.g. ITU / Theatre capacity)

NONE-we are not doing liver transplantation in my centre

Not applicable

Other (please specify)

Q25. What is your main expectation from new machine perfusion technology (or what would you want to be improved) – please select the most important one from the list.

Answer Choices

Improve donor organ quality, reduce ischemia / reperfusion injury and prevent complications

Positive impact on logistical issues (e.g. bridge lack of ITU / Theatre capacity)

Easy to implement in clinical practice

Cost effective

Q26. What is your second most important expectation from new machine perfusion technology (or what would you want to be improved) – please select a different one from the list.

Answer Choices

Improve donor organ quality, reduce ischemia / reperfusion injury and prevent complications

Positive impact on logistical issues (e.g. bridge lack of ITU / Theatre capacity)

Easy to implement in clinical practice

Cost effective

Q27. What would you consider as acceptable price for one perfusion device ?

Answer Choices

0 - 20`000 Euro

> 20`000 - 50`000 Euro

> 50`000 - 80`000 Euro

> 80`000 - 120`000 Euro

> 120`000 - 150`000 Euro

> 150`000 Euro

Q28. What would you consider as acceptable price for one disposable for organ perfusion (one organ perfusion)?

Answer Choices

0 - 1`000 Euro

> 1`000 - 2`500 Euro

> 2`500 - 5`000 Euro

> 5`000 - 10`000 Euro

> 10`000 - 15´000 Euro

> 15`000 - 20`000 Euro

>20`000 - 30`000 Euro

> 30`000 Euro

Q29. How should the perfusion device be financed ?

Answer Choices

Direct payment of perfusion device

Loan of device with higher costs for the disposable

Rent

Rent to buy

Lease with option to buy

Other (please specify)

Q30. Which entity should finance machine perfusion technology ?

Answer Choices

Government or public national health system

Hospital

Hospital through recipient insurance

Privately financed by organ recipient

Regional or national transplantation society

Research Grant

Charity

Q31. Considering the available research data do you think machine perfusion technology should be integrated into routine clinical practice with full commissioning ?

Answer Choices

Yes

No

Q32. Are there relevant limitations to implement machine perfusion technology in your country or centre ?

Answer Choices

Yes

No

Q33. Please select the main reason that precludes the implementation of machine perfusion in

your centre.

Answer Choices

Lack of knowledge on machine perfusion technology

I consider machine perfusion NOT superior to standard cold storage

Lack of staff in general

Lack of trained staff

No or limited funding opportunities

Logistical issues (e.g. device transport)

In my centre there is full support to implement machine perfusion technology.

Not applicable

Q34. Do you think machine perfusion technology increases the utilisation of riskier organs ?

Answer Choices

Yes

No

Q35. Which organ perfusion modality would be the best to increase the utilisation of riskier organs ?

Answer Choices

Normothermic regional perfusion (NRP)

Hypothermic machine perfusion (HMP)

Hypothermic oxygenated perfusion (additional perfusate oxygen, HOPE, D-HOPE, HMP-O2)

Normothermic machine perfusion (NMP)

None

Other (please specify)

Q36. For what type of donors or LIVERS would you consider machine perfusion routinely ?

Answer Choices

Any donor liver (e.g. any age or any quality, including any DBD, DCD, ...)

Advanced donor age

Livers from extended criteria donors (marginal grafts)

Livers from donation after circulatory death (DCD) donors

Steatotic livers

NONE-we are not doing liver transplantation in my centre

Other (please specify)

Q37. Which organ perfusion modality would be the best to protect liver recipients from ischemic cholangiopathy (incl. ITBL, NAS, IC) ?

Answer Choices

Normothermic regional perfusion (NRP)

Hypothermic machine perfusion (HMP)

Hypothermic oxygenated perfusion (additional perfusate oxygen, HOPE, D-HOPE, HMP-O2)

Normothermic machine perfusion (NMP)

None

NONE-we are not doing liver transplantation in my centre

Other (please specify)

Q38. Do you think normothermic perfusion is equally protective from reperfusion injury if applied instead of cold storage (with device transport) or after cold storage in the recipient centre ?

Answer Choices

Yes

No

Q39. At your centre do you base the decision to accept or decline an organ for transplantation on specific viability criteria assessed during machine perfusion ?

Answer Choices

Yes

No

Not applicable

Q40. Please state which main viability parameter / criteria you follow during organ machine perfusion.

Answer Choices

Organ parameter (e.g. macroscopy, perfusion quality, soft organ, bile production,...)

Perfusion parameter (e.g. flow, pressure, resistance,...)

Markers of organ injury measured in perfusate (e.g. lactate, enzymes,...)

Markers of organ function, representative for a specific cell compound (e.g. mitochondria,...)

We do NOT consider parameters measured during machine perfusion for viability assessment.

Not applicable

Q41. Do you consider perfusate Lactate (measured during machine perfusion) as reliable parameter to decide to accept a liver for transplantation or not ?

Answer Choices

Yes

No

No - we combine Lactate with other parameters

I don`t know

Q42. Please add further comments or suggestions.
